# Artificial Intelligence in Coronary Computed Tomography Angiography: From Anatomy to Prognosis

**DOI:** 10.1155/2020/6649410

**Published:** 2020-12-16

**Authors:** Giuseppe Muscogiuri, Marly Van Assen, Christian Tesche, Carlo N. De Cecco, Mattia Chiesa, Stefano Scafuri, Marco Guglielmo, Andrea Baggiano, Laura Fusini, Andrea I. Guaricci, Mark G. Rabbat, Gianluca Pontone

**Affiliations:** ^1^Centro Cardiologico Monzino, IRCCS, Milan, Italy; ^2^Division of Cardiothoracic Imaging, Nuclear Medicine and Molecular Imaging, Department of Radiology and Imaging Sciences, Emory University, Atlanta, GA, USA; ^3^Department of Cardiology, Munich University Clinic, Ludwig-Maximilians-University, Munich, Germany; ^4^Department of Internal Medicine, St. Johannes-Hospital, Dortmund, Germany; ^5^Division of Interventional Structural Cardiology, Cardiothoracovascular Department, Careggi University Hospital, Florence, Italy; ^6^Institute of Cardiovascular Disease, Department of Emergency and Organ Transplantation, University Hospital “Policlinico Consorziale” of Bari, Bari, Italy; ^7^Loyola University of Chicago, Chicago, IL, USA; ^8^Edward Hines Jr. VA Hospital, Hines, IL, USA

## Abstract

Cardiac computed tomography angiography (CCTA) is widely used as a diagnostic tool for evaluation of coronary artery disease (CAD). Despite the excellent capability to rule-out CAD, CCTA may overestimate the degree of stenosis; furthermore, CCTA analysis can be time consuming, often requiring advanced postprocessing techniques. In consideration of the most recent ESC guidelines on CAD management, which will likely increase CCTA volume over the next years, new tools are necessary to shorten reporting time and improve the accuracy for the detection of ischemia-inducing coronary lesions. The application of artificial intelligence (AI) may provide a helpful tool in CCTA, improving the evaluation and quantification of coronary stenosis, plaque characterization, and assessment of myocardial ischemia. Furthermore, in comparison with existing risk scores, machine-learning algorithms can better predict the outcome utilizing both imaging findings and clinical parameters. Medical AI is moving from the research field to daily clinical practice, and with the increasing number of CCTA examinations, AI will be extensively utilized in cardiac imaging. This review is aimed at illustrating the state of the art in AI-based CCTA applications and future clinical scenarios.

## 1. Introduction

Coronary computed tomography angiography (CCTA) represents an excellent tool for the evaluation of patients with suspected stable coronary artery disease (CAD) [1–6]. There is strong evidence in the literature that CCTA can accurately rule out the presence of CAD, having a positive impact in terms of prognosis and cost [7–11].

CCTA represents an important step in clinical management of patients with suspected CAD; however, it is important to keep in mind that the majority of CCTA results in no evidence of significant CAD [12, 13]. Furthermore, the presence of obstructive CAD on CCTA is not always associated with the development of myocardial ischemia [14].

The application of artificial intelligence (AI) in cardiac radiology is aimed at facilitating the management of patients with suspected CAD ranging from diagnosis to prognostic stratification [15]. In particular, the application of AI can be helpful in reducing the time of image analysis and rule out patients without evidence of significant disease that may benefit from medical therapy [16]. Furthermore, it can be helpful for detection of myocardial ischemia [17]. In terms of prognostic stratification, AI may play a promising role, identifying algorithms that can stratify the risk of major adverse cardiovascular events (MACE) with high accuracy [18].

## 2. Basic Concept of AI in Clinical Medicine

The AI industry has seen massive growth in a variety of fields in the past decade, with the field of medicine not being an exception. The basis of AI is mathematics and computer science with the three main pillars being (1) big data, (2) high performance computing infrastructure, and (3) algorithm development. The exponential growth in digital storage capabilities, data collection systems, and computing power enabled AI applications in a wide variety of fields. The current digital era leads to an increased amount of information, which is beneficial to the development of AI algorithms. The technological developments make it possible to develop algorithms that are able to deal with the large amount of data and complexity typical of the digital era we live in.

With AI currently entering the medical field, early stage applications have mainly focused on automatization of medical tasks; more recently, the focus has shifted towards prognostication and risk prediction. Many studies investigate the potential role of AI in supporting clinicians in their day-to-day tasks, assisting in workflow optimization, quantification, diagnosis and prognostication, and reporting. However, many clinical AI applications are currently only used in a research setting and are far from being implemented into clinical practice. There are examples of successful AI implementation [19]. The Data Science Institute of the American College of Radiology has published a list of all FDA cleared AI algorithms for radiology purposes [20] with their state of validation and clinical use. However, there are also examples of applications that are not ready for clinical utilization [21, 22]. For example, Zech et al. assessed how well convolutional neural networks (CNN) generalized across three hospital systems for a simulated pneumonia screening task. They found that their evaluated CNN performed systematically worse on unseen data from different hospitals compared to the training set. In addition, they reported that the CNN identified disease burden within hospital system and department, which may confound predictions [21]. A thorough clinical validation is essential for the acceptance and implementation of AI into clinical practice [22]. A study by Kim et al. evaluated the validation of AI algorithms reported in AI research papers from all medical fields, including radiology, dermatology, and pathology. They reported that only 6% of all studies used external validation to assess AI algorithm performance [22]. Since then, several guidelines have been published to improve the validation process of medical AI applications [23, 24]. Recently, we have seen an increase in publications that externally validate industry developed AI algorithms [25, 26]. In addition, regulations and guidelines regarding protection of patient privacy and cybersecurity are also needed. Creating awareness and increasing basic AI knowledge for clinicians are an essential step to promote wide AI acceptance among physicians and patients.

The European commission released a white paper on AI in February 2020, including statements on the use of AI for medical purposes [27, 28]. They state that current EU regulations already provide a high level of protection through medical device laws and data protection laws; however, they proposed to add specific regulations including requirements of training data, record-keeping of used datasets, transparency, robustness and accuracy, and human oversight. The US counterpart, the U.S. Food and Drug Administration, also released statements regarding the use of medical AI. While application for medical assistance, such as quantification applications, only requires a proof of equivalence to other software (510(k)) [20, 29], application for clinical interpretation of medical data is a more elaborate FDA approval (PMA).

**Figure 1:**
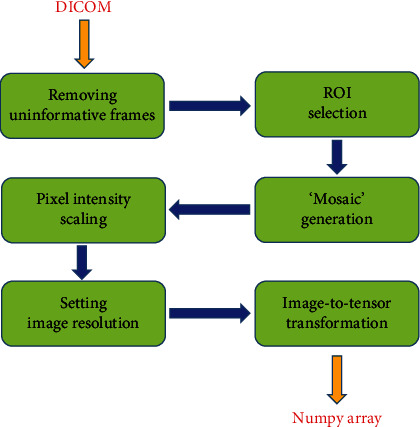
Streamline used for the development of images useful for DL algorithm starting from DICOM images.

Besides the legal framework for medical AI, there are some ethical considerations that will play a key role [30]. With the use of medical data, issues such as gender, race, or economical discrimination due to underrepresentation in the training populations should be discussed and evaluated. In addition, AI-based risk prediction and prognostication can be used to limit the choice and coverage of healthcare insurance in certain groups of patients or can affect important life choices. Like every new technology in the medical field, it is imperative to learn how to balance the benefits and risks associated with a broad AI implementation and how to democratize AI and make sure that everybody can benefit equally from its use. The Joint European and North American Multisociety task force discusses these issues in detail, emphasizing that more research is needed on the implementation of AI into clinical practice [31].  Figure 1 shows the process of DICOM images elaboration for development of DL algorithm.

## 3. AI Application for the Evaluation of Coronary Artery Stenosis

The grading and coronary segments involved with obstructive of CAD have been associated with a worse prognosis [32].

Often assessment of CAD stenosis is time consuming, requiring multiplanar reconstruction selection of the best phase in the cardiac cycle for a correct assessment of coronary arteries and depends on the experience of the reader [15]. After the CTA analysis, results may be reported extensively in the report following the guidelines of SCCT [33] or in a structured patient-based approach identifying a specific CAD-RADS grading [34].

Zreik et al. developed a 3D CNN that was able to characterize the plaque and evaluate the grading of stenosis [35]. The authors developed two models; the first one analyzed the performance of the algorithm to differentiate patients with/without obstructive CAD demonstrating a per-segment, vessel, and patient accuracy of 0.94, 0.93, and 0.85, respectively [35]. The second model was developed for identification of no stenosis, no significant stenosis, and significant stenosis; the second model showed a per-segment, vessel, and patient accuracy of 0.80, 0.76, and 0.75, respectively [35].

Kang et al. developed an AI technique based on a two-step algorithm with a vector machine that was useful for the evaluation of CAD stenosis [36]. On a population of 42 patients acquired with dual source CT, the algorithm was able to identify the grade of stenosis in one second with a sensitivity, specificity, and accuracy in the proximal and midsegments of 93%, 95%, and 94%, respectively [36].

Yoneyama et al. evaluated the possibility to identify the grading of coronary stenosis and its impact in terms of ischemia using a cohort of patients who underwent CCTA and perfusion single photon emission computed tomography (SPECT) [37]. The authors focused on the application of an artificial neural network (ANN) with hybrid imaging obtained by the combination of CCTA and myocardial perfusion SPECT [37]. Using this algorithm, the specificity, sensitivity, and accuracy to identify coronary artery stenosis >70% were 31%, 78%, and 67%, respectively [37].

Van Hamersvelt et al. developed an algorithm of AI that evaluated the presence of significant CAD using a combined approach of AI that analyzes the myocardium and compared it with invasive FFR [38]. They found that a combined approach was able to identify hemodynamically significant CAD with an AUC of 0.76.

Two studies developed an automated approach of CADRADS in clinical practice [16, 39].

**Figure 2:**
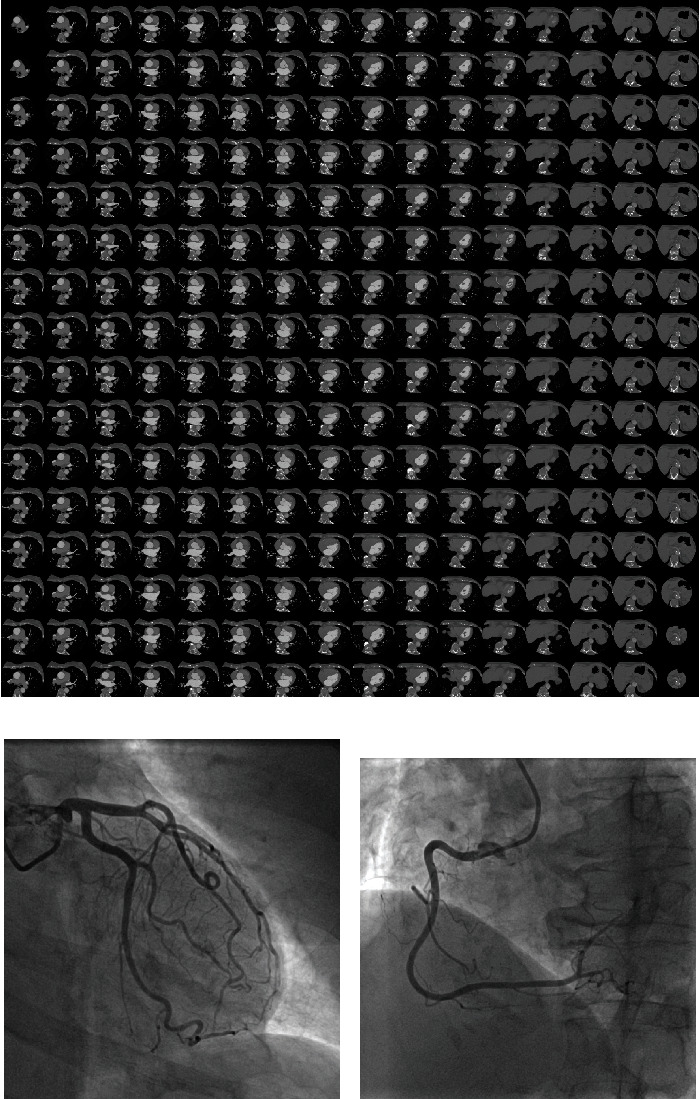
A 54-year-old female patient scheduled for invasive coronary angiography. Reconstruction for CAD-RADS algorithm is shown in (a). The algorithm provides a CAD − RADS = 0. This finding was confirmed on coronary angiography that shows no disease in the left coronary artery (b) and right coronary artery (c).

Muscogiuri et al. evaluated the impact of a new deep learning algorithm based on CNN for the classification of CAD-RADS in a cohort of 288 patients who underwent CCTA for a clinical indication [16]. The time of analysis and accuracy for each of the following was extrapolated: Model A (CAD-RADS 0 vs. CAD-RADS 1-2 vs. CAD-RADS 3, 4, 5), Model 1 (CAD-RADS 0 vs. CAD − RADS > 0), and Model 2 (CAD-RADS 0-2 vs. CAD-RADS 3-5) [16]. The sensitivity, specificity, negative predictive value, positive predictive value, and accuracy of the models compared to humans were the following: Model A, 47%, 74%, 77%, 46%, and 60%; Model 1, 66%, 91%, 92%, 63%, and 86%; and Model 2, 82%, 58%, 74%, 69%, and 71% [16]. The average time of analysis of CNN was significantly shorter compared to humans, with an average time of analysis around 104 seconds [16]. This study highlights the possibility to have an automatic discrimination between patients with CAD − RADS > 0 with a high diagnostic accuracy and short time. This is an important finding if we assume an increased number of CCTA scans in the future, many of which may not show CAD [12, 13]. A representative case showing the application of the CAD-RADS software for detection of AI is shown in Figure 2.

Another important application of automatic CAD-RADS classification was shown by Huang et al. [39]. The authors classified CAD-RADS using a deep learning algorithm and subsequently correlated the results with the presence of arterial breast calcification. The authors showed that the presence of high grade CAD-RADS was closely associated with increased presence of breast arterial calcification [39]. This finding is important because the assessment of breast arterial calcification in screening for breast cancer can be utilized for early identification of patients with CAD.

## 4. AI for Evaluation of Plaque Analysis

### 4.1. Calcium Score

Coronary Artery Calcium Score (CACS) is an independent predictor of adverse cardiovascular events [40–42].

CT images for the evaluation of calcium score are often acquired using an ECG-gated, no contrast technique and segmented calculating a calcium volume, and mass obtaining a specific value of calcium score [43]. Currently, CACS is performed by semiautomatic segmentation and despite a time consuming approach is still the gold standard [44].

The evaluation of CACS using an AI algorithm can definitely speed up the time of reporting.

One of the first articles describing the evaluation of CACS using an algorithm of AI was developed by Isgum et al. [45]. The authors analyzed the impact of the automated algorithm on ECG-gated, noncontrast images, and identified coronary calcification in 73.8% of cases and 93.4% of cases was correctly classified in the respective risk group [45].

Sandsted et al. evaluated the performance of an AI algorithm for the evaluation of CACS compared to semiautomated CACS [46]. The authors found a Spearman's rank correlation coefficient for Agatston Score, Calcium Volume Score, and Calcium Mass Score between the AI algorithm and semiautomatic approach of 0.935, 0.932, and 0.934, respectively, while the intraclass correlations were 0.996, 0.996, and 0.991, respectively, [46].

Despite CACS traditionally being evaluated using ECG-gated scans, recently, Takx et al. analyzed the impact of AI for evaluation of CACS in non-ECG-gated and noncontrast images acquired in a cohort of patients undergoing a CT for lung cancer screening [47]. In a cohort of 1793 patients, the authors analyzed the impact of an AI algorithm for detection of CACS. Despite a small percentage of the population (44 patients representing the 2.5%) being excluded from the study due to image quality, the authors found good reliability with a weighted *k* of 0.85 for Agatston risk score between the automated and reference scores [47]; however, an underestimation in terms of volume of calcium was observed in the automatic segmentation compared to manual segmentation [47].

The combination of CACS analysis and lung cancer screening can be a powerful combination in clinical practice to identify patients that may benefit from therapy.

Wolterink et al. described the application of an automated algorithm for the evaluation of CAC in 250 patients who underwent CCTA [48]. The authors described a supervised approach and developed a CNN algorithm that was able to identify CAC with a sensitivity of 0.72 and an interclass correlation of 0.94 between CAC derived from CCTA and standard evaluation of CAC [48]. This approach may lead to radiation dose reduction.

Finally, Van Velzen et al. evaluated calcium scores from different CT without contrast [49]. 7240 examinations were analyzed from PET attenuation CT images and CT of the chest demonstrating an intraclass correlation coefficient ranging from 0.79 to 0.97 when compared with manual segmentation [49]. An approach that is independent of ECG-gated acquisition, allowing for automated analysis, represents an important tool.

### 4.2. Plaque Phenotype

Assessment of plaque composition is extremely important in CCTA reporting; indeed, identification of fibrous or calcified plaques can be extremely important for patient management [50]. Presence of calcified plaques is associated with better outcome compared to fibrous plaques, especially in the presence of high-risk plaque characteristics [51].

The application of AI can facilitate and speed up the analysis of CCTA providing accurate information on plaque analysis in a relative short time.

Zreik et al. developed an algorithm that was able to identify the plaque morphology and severity of stenosis [35]. From a sample size of 95 patients, the authors developed an AI approach based on 3D CNN that extrapolated the characteristics of plaque along the coronary arteries. Subsequently, the images were tested on a smaller cohort composed of 65 patients showing an accuracy of 0.85 for differentiation between plaque and no plaque while the accuracy for differentiation between different types of plaque was 0.77 [35].

Another application of AI for identification of different plaque types was developed by Dey et al. The authors developed an algorithm that automatically differentiated calcified plaque (*r*: 0.88) and noncalcified plaque (*r*: 0.98) with a good correlation compared to manual segmentation [52].

A different, combined approach of radiomics and machine learning (ML) for the evaluation of plaque characteristics has been demonstrated to characterize plaque [53]. Using radiomics, from standard images, it is possible to obtain several parameters that can constitute the fingerprinting of a plaque.

Kolossvary et al. evaluated the radiomic features of plaques showing napkin ring sign (NRS) which has been associated with poor outcome [54]. The authors describe the parameter called “short-run low-gray-level emphasis”; this parameter was able to identify plaque with NRS with a better accuracy (AUC 0.89) compared to mean plaque attenuation (AUC 0.75), the latter used in standard clinical practice [54].

An ML approach can identify the presence of thin cap fibroatheroma (TCFA) overcoming the technical limitation of CCTA [53]. In particular, Masuda et al. analyzed the application of an ML histogram for the identification of fibrous and fatty or fibrous-fatty plaques compared to IVUS showing an accuracy of 0.92, while standard parameters showed an accuracy of 0.83 [55].

### 4.3. AI for the Assessment of Ischemia: CT-Derived Fractional Flow Reserve and CT Perfusion

Recent research and development in AI has been applied in multiple potential applications of cardiac CT-derived myocardial ischemia assessments. Most software applications herby deal with CT-derived fractional flow reserve (FFR) for the detection of hemodynamically significant CAD. Only few studies of AI applications using CT perfusion have been published so far. In terms of CT-FFR, ML solutions have been provided by only one vendor [56, 57]. However, this approach is for research purposes only. More recently, a commercially available software application (DeepVessel FFR) has been introduced by Keya Medical (Beijing, China) [58].

**Figure 3:**
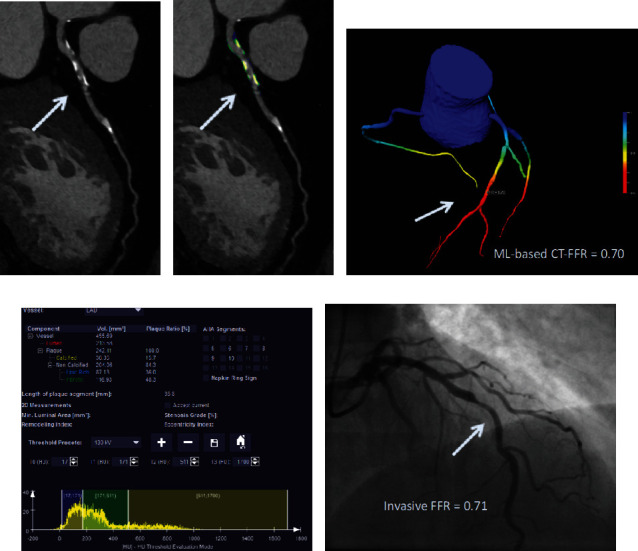
Coronary CT angiography in a 54-year-old man without known coronary artery disease. (a) Automatically generated curved multiplanar reformations showing >50% stenosis of the proximal LAD (arrow). (c) 3-Dimensional color-coded mesh shows a CT-FFR value of 0.70, indicating ischemia of the underlying stenosis (arrow). (b, d) Color-coded automated plaque assessment of the lesion demonstrating the predominantly calcified composition of the atherosclerotic atheroma. (e) Invasive coronary angiography confirms obstructive stenosis of the LAD (arrow) with an FFR of 0.70.

ML-based CT-FFR employs a multilayer neural network framework that was trained and validated offline against the former CFD approach by using a virtual dataset of 12.000 synthetic 3D coronary models [56]. The clinical validation of the ML approach has been conducted in one multicenter trial and several single-center studies in relation to CCTA and invasive coronary angiography (ICA) assessing lesion-specific ischemia. The MACHINE registry (Diagnostic Accuracy of a Machine-Learning Approach to Coronary Computed Tomographic Angiography - Based Fractional Flow Reserve: Result from the MACHINE Consortium) investigated ML-based CT-FFR in 351 patients with 525 vessels from 5 sites in Europe, Asia, and the United States [57]. The diagnostic accuracy of ML-based CT-FFR was significantly better when compared to that of CCTA (ML CT-FFR 78% vs. cCTA 58%). Likewise, the AUC for identifying hemodynamically significant CAD was superior for ML-based CT-FFR (AUC: 0.84) in comparison to that of CCTA alone (AUC: 0.69, *p* < 0.05). In accordance with the results of the MACHINE registry, several single-center studies have evaluated the diagnostic performance of ML-based CT-FFR, reporting sensitivities and specificities ranging from 79% to 82% and 91% to 94%, respectively [59, 60]. ML-based CT-FFR has also proven its feasibility in coronary calcification. A recent study by Tesche et al. [61] investigated the impact of coronary calcifications on the accuracy of ML-CT-FFR. The authors reported a good but statistically significant different diagnostic performance of ML CT-FFR in heavily calcified vessels in comparison to low-to intermediate ranges of calcifications (AUC: 0.71 vs. 0.85, *p* = 0.04). Another substudy of the MACHINE registry assessed the impact of gender on the diagnostic accuracy of ML CT-FFR with no significant difference in the AUCs in men when compared to that of women (AUC: 0.83 vs. 0.83, *p* = 0.89) [62]. Overall, ML-based CT-FFR provides high diagnostic accuracy for the assessment of lesion-specific ischemia. A representative case is shown in Figure 3.

Only few studies have assessed the use of AI for CT perfusion. However, CT perfusion offers a field with great potential for the application of AI especially for automated identification of perfusion defects and myocardial segmentation. Preliminary results have demonstrated an AUC of 0.73 by using different ML approaches for automated segmentation and delineation of the left ventricle when compared to manual segmentation by an expert reader [63]. In another investigation, Han and colleagues [64] used a gradient boosting classifier for supervised ML in resting myocardial perfusion CT for the identification of lesion-specific ischemia. The authors showed a diagnostic accuracy, sensitivity, and specificity of 68%, 53%, and 85% of CTP added to cCTA stenosis > 70% for predicting hemodynamically significant CAD.

## 5. AI in CCTA Prognostication

Focusing on outcome, there are several manuscripts that show the impact of CAD depicted on CCTA and prognosis [8, 65]. An algorithm based on AI can improve risk stratification based on standard clinical parameters.

Motwani et al. evaluated the impact of an ML algorithm for prognostic stratification in a large cohort of 10030 patients with follow-up of 5 years and an endpoint of mortality [66]. A total of 25 clinical parameters and 44 CCTA parameters were evaluated for a correct assessment of mortality that occurred in seven hundred and forty-five patients [66]. The ML algorithm was superior compared to Framingham Risk Score (FRS) or CCTA severity risk scores with an area under curve (AUC) of 0.79 while FRS showed an AUC of 0.61, segment stenosis score of 0.64, segment involved score of 0.64, and modified Duke index of 0.62 [66].

Van Rosendael et al. developed a model for risk stratification based on a population from the CONFIRM registry [67]. The primary endpoint was a composite of myocardial infarction and death, and the algorithm was able to predict the primary endpoint with an AUC of 0.77 versus the other scores that ranged from 0.65 to 0.70.

Tesche et al., in a small cohort of patients, developed an AI algorithm for risk stratification in patients who underwent CCTA with follow-up of 5.4 years [18]. The authors found that an ML approach showed an AUC of 0.96 for MACE, higher compared to Agatston calcium score (AUC: 0.84), segment involved score (AUC: 0.88), and segment stenosis score (AUC: 0.89).

## 6. Future Perspectives

In CCTA, the role of AI may be important for further radiation dose reduction [68] without impairment of image quality and help in CCTA reporting, evaluation of CAD burden, myocardial ischemia, and assessment of prognosis [15] (Table 1).

Human interpretation, despite their experience, is still prone to fatigue. Furthermore, the time of training of expert readers requires years of experience. The application of AI in CCTA will not substitute the cardiac radiologist; rather, AI will represent a helpful tool for reporting and prognostic stratification. Indeed, following the ESC guidelines [7], over the next few years, the requests for CCTA will increase. Therefore, a helpful tool that can decrease the time of CCTA analysis should be embraced.

Furthermore, CCTA analysis is moving toward a model of precision medicine. The analysis of coronary stenosis grading is not sufficient alone. A comprehensive CCTA report needs to provide information regarding characterization of plaque and its hemodynamical effect; furthermore, the joint evaluation of clinical parameters can be helpful to stratify the patients in terms of worse outcome and can be helpful for individual treatment plans.

**Figure 4:**
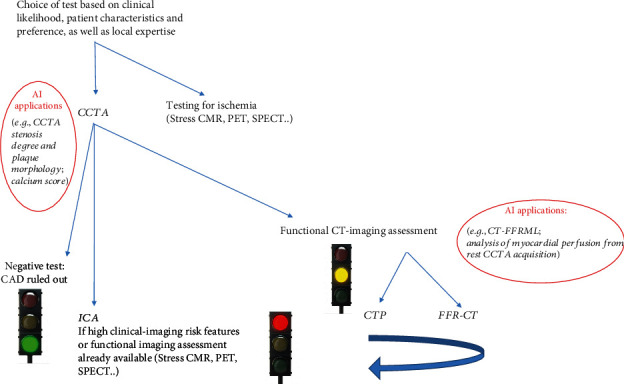
Application of AI on CCTA in the clinical setting. First, CCTA images are processed using an AI algorithm; subsequently, the patients can be further classified in three groups: patients without obstructive CAD, patients that need invasive coronary angiography, and patients with stenosis that could benefit from functional imaging. In the cohort of patients classified to functional imaging such as CT perfusion or CT-FFR, an algorithm of AI can be applied in order to speed up the process.

It is plausible that an algorithm will be composed for automatic analysis of CCTA images followed by detection of myocardial ischemia (Figure 4). Subsequently, the final results of CCTA will be evaluated according to the clinical parameters with an AI algorithm in order to obtain a patient-based risk profile.

Strict legislation focused on the application of AI in cardiac imaging will be necessary to clarify the medico-legal aspects of the AI algorithm application. Furthermore, the development of an AI algorithm implies the analysis of a large amount of data; this aspect is extremely important if we consider the legal aspects due to privacy.

All these aspects need to be clarified in the future before we consider the application of AI in routine clinical practice.

## 7. Conclusion

In the future, AI will be integrated in the CCTA workflow. AI applications will greatly benefit CCTA practice reducing the reporting time and providing a more accurate quantitative-based approach to CAD management, moving the entire field in the direction of precision-based medicine. However, before we can widely implement AI solutions in our clinical practice, we need to carefully validate the algorithms in the light of standards for good medical practice and new medical device utilization and carefully address possible issues on data protection, legal framework, and ethical principles.

## Figures and Tables

**Table 1:** Impact of AI in CCTA.

Task	Accuracy
Coronary artery stenosis	*++/+++*
Coronary calcium	*++*
Plaque phenotype	*++*
Detection of ischemia	*++/+++*
Prognosis	*++/+++*

AI: artificial intelligence; CCTA: coronary computed tomography angiography.

## Data Availability

Data from our manuscript are obtained from the article cited in the manuscript.
